# Checklist of rodents and insectivores of the Mordovia, Russia

**DOI:** 10.3897/zookeys.1004.57359

**Published:** 2020-12-16

**Authors:** Alexey V. Andreychev, Vyacheslav A. Kuznetsov

**Affiliations:** 1 Department of Zoology, National Research Mordovia State University, Bolshevistskaya Street, 68. 430005, Saransk, Russia National Research Mordovia State University Saransk Russia

**Keywords:** Insectivores, Mordovia, rodents, spatial distribution

## Abstract

A list of 40 species is presented of the rodents and insectivores collected during a 15-year period from the Republic of Mordovia. The dataset contains more than 24,000 records of rodent and insectivore species from 23 districts, including Saransk. A major part of the data set was obtained during expedition research and at the biological station. The work is based on the materials of our surveys of rodents and insectivorous mammals conducted in Mordovia using both trap lines and pitfall arrays using traditional methods.

## Introduction

There is a need to review the species composition of rodents and insectivores in all regions of Russia, and the work by Tovpinets et al. (2020) on the Crimean Peninsula serves as an example of such research. Studies of rodent and insectivore diversity and distribution have a long history, but there are no lists for many regions of Russia of rodent and insectivorous species. Lists of species have been updated for a few regions, with some species excluded and others added. The Republic of Mordovia is one of these regions, where eminent theriologists (S.I. Ognev, S.S. Turov, L.G. Morozova-Turova, I.I. Barabash-Nikiforov, L.P. Borodin, M.N. Borodina, P.L. Borodin) once worked. The inventory of the mammalian fauna of Mordovia resumed at the beginning of the 21^st^ century as part of dissertation research and continues to this day. Over this period, not only has the species composition of the region changed but also the status of many species.

The Mordovian fauna is heterogeneous and consists of four different ecological and faunal complexes of species–taiga, coniferous and broad-leaved forests, and steppe–which are widely distributed in several natural areas (Andreychev 2020).

Here, we publish a checklist of rodent and insectivore records across the Republic of Mordovia. This checklist was based on comprehensive surveys of small mammals carried out from 2006 to 2020.

Insectivores are represented in Mordovia by 12 species belonging to three families.

**Family Erinaceidae Fischer, 1814**

1. Northern white-breasted hedgehog, Erinaceus roumanicus Barrett-Hamilton, 1900
2. West European hedgehog, Erinaceus europaeus Linnaeus, 1758

**Family Talpidae Fischer, 1814**

1. European mole, Talpa europaea Linnaeus, 1758
2. Russian desman, Desmana moschata (Linnaeus, 1758)

**Family Soricidae Fischer, 1814**

1. Eurasian common shrew, Sorex araneus Linnaeus, 1758
2. Laxmann’s shrew, Sorex caecutiens Laxmann, 1788
3. Least shrew, Sorex minutissimus Zimmermann, 1780
4. Taiga shrew, Sorex isodon Turov, 1924
5. Eurasian pygmy shrew, Sorex minutus Linnaeus, 1766
6. Eurasian water shrew, Neomys fodiens (Pennant, 1771)
7. Southern water shrew, Neomys anomalus Cabrera, 1907
8. Lesser white-toothed shrew, Crocidura suaveolens (Pallas, 1811)

Rodents are represented by 29 species belonging to eight families.

**Family Sciuridae Fischer, 1817**

1. Red squirrel, Sciurus vulgaris Linnaeus, 1758
2. Spotted suslik, Spermophilus suslicus Güldenstaedt, 1770
3. Bobak marmot, Marmota bobak (Müller, 1776)

**Family Castoridae Hemprich, 1820**

1. Eurasian beaver, Castor fiber Linnaeus, 1758

**Family Gliridae Thomas, 1897**

1. Forest dormouse, Dryomys nitedula (Pallas, 1779)
2. Garden dormouse, Eliomys quercinus (Linnaeus, 1766)
3. Fat dormouse, Glis glis (Linnaeus, 1766)
4. Hazel dormouse, Muscardinus avellanarius (Linnaeus, 1758)

**Family Sminthidae Brandt, 1855**

1. Northern birch mouse, Sicista betulina (Pallas, 1779)

**Family Allactagidae Vinogradov, 1925**

1. Great jerboa, Allactaga major (Kerr, 1792)

**Family Spalacidae Gray, 1821**

1. Greater mole rat, Spalax microphthalmus Güldenstaedt, 1770
2. Family Cricetidae Fischer, 1817
3. Bank vole, Myodes glareolus (Schreber, 1780)
4. Northern red-backed vole, Myodes rutilus (Pallas, 1779)
5. European water vole, Arvicola amphibius (Linnaeus, 1758)
6. Root vole, Microtus oeconomus (Pallas, 1776)
7. Gray dwarf hamster, Cricetulus migratorius (Pallas, 1773)
8. Common hamster, Cricetus cricetus (Linnaeus, 1758)
9. Muskrat, Ondatra zibethicus (Linnaeus, 1766)
10. Steppe lemming, Lagurus lagurus Pallas, 1773
11. Common vole, Microtus arvalis (Pallas, 1779)
12. East European vole, Microtus rossiaemeridionalis Ognev, 1924
13. Field vole, Microtus agrestis (Linnaeus, 1761)
14. European pine vole, Microtus subterraneus (de Selys-Longchamps, 1836)

**Family Muridae Illiger, 1811**

1. Striped field mouse, Apodemus agrarius (Pallas, 1771)
2. Pygmy wood mouse, Apodemus uralensis (Pallas, 1811)
3. Yellow-necked wood mouse, Apodemus flavicollis (Melchior, 1834)
4. Harvest mouse, Micromys minutus (Pallas, 1771)
5. House mouse, Mus musculus Linnaeus, 1758
6. Norway rat, Rattus norvegicus (Berkenhout, 1769)

Genetic studies of two similar species, *Microtus arvalis* and *M. rossiaemeridionalis*, have not been conducted in the region. Approaches and criteria for differentiation of two similar species, *Erinaceus roumanicus* and *E. europaeus*, have been applied for a number of specific morphological and craniometric characteristics (Zaitsev 1984; Frost et al. 1991). First, *E. roumanicus* has a patch of white hair on its belly. And *E. europaeus* has no white hair on its belly. Differences in the skull are also apparent; in *E. roumanicus*, the premaxillary-maxillary suture has one or two inflections (in *E. europaeus* it is smooth), the length of the premaxillary-nasal suture does not exceed 9.0 mm (in *E. europaeus >*9.0 mm), and the maximum length of the nasal bones in their back part is greater than or equal to 3.0 mm (*E. europaeus* <3.0 mm) (Zaitsev 1984; Frost et al. 1991; Zaitsev et al. 2014). The color of the needles of these two species can serve as a criteria for their differences. Six species (*Eliomys quercinus*, *Cricetulus migratorius*, *Lagurus lagurus*, *Myodes rutilus*, *Microtus subterraneus*, and *Neomys anomalus*) reported from the Republic of Mordovia were not detected during our surveys. However, these species were captured by our colleagues, either long ago or even in the last year, and most of them (*E. quercinus*, *C. migratorius*, *L. lagurus*, and *M. rutilus*) were recorded in the Mordovian state nature reserve, Temnikovsky district (Borodina et al. 1970). *Microtus subterraneus* has recently been found in Mordovia (Kirillova et al. 2019). This record represents the easternmost occurrence of of this species. Previously, this underground vole was recorded in neighboring regions of Mordovia, namely near the village of Zhelannoe in Ryazan region and from Zametchinsky district in Penza region. This species is rare and included in Red Data Books of several Russian regions, including the Leningrad, Tver, Penza, Moscow, Pskov reion, and Novgorod regions. The appearance of a new species for Mordovia can be explained by its expansion into new territories. This is confirmed by the new record of this species from the Smolensk region (Belyaev 2020). In addition, a species atypical of the Mordovian fauna, *Neomys anomalus*, is now known (Borodin 2013).

From an ecological perspective, mesophilous species comprise the largest group, which includes 26 species. Some xerophilous species (*M. bobak*, *Sp. suslicus*, *Al. major*, *Sp. microphthalmus*, *Cr. migratorius*, *Cr. cricetus*, *L. lagurus*, and *M. minutus*) occur only in steppe habitats. Only in the steppe areas of Mordovia are there concentrated populations of *S. microphthalmus* (Andreychev 2018, 2019) and *M. bobak* (Andreychev et al. 2015). Populations of *S. microphthalmus* in Mordavia are vulnerable, as in other parts of its range (Zagorodniuk et al. 2018). Grazing is important here for *M. bobak*, as has been shown for Ukraine (Rashevska and Semeniuk 2015; Tokarsky 2015; Savchenko and Ronkin 2018).

Dendrophile rodents are represented by only seven species: *D. nitedula*, *G. glis*, *M. avellanarius*, *E. quercinus*, *S. vulgaris*, *A. flavicollis*, and *A. uralensis*. Of these species, the most studied in the region are *D. nitedula* (Andreychev and Boyarova 2020; Andreychev and Kiyaykina 2020), *A. flavicollis*, and *A. uralensis* (Andreychev and Kuznetsov 2012).

Thirteen species are associated with human settlements, *C. suaveolens*, *E. roumanicus*, *S. minutus*, *S. araneus*, *S. isodon*, *C. cricetus*, *M. glareolus*, *M. arvalis*, *A. agrarius*, *A. uralensis*, *A. flavicollis*, *M. musculus*, and *R. norvegicus*, and these have been repeatedly been recorded in residential areas. However, only three species, *M. musculus*, *R. norvegicus*, and *C. suaveolens*, are truly commensal.

The rodent and insectivore fauna of Mordovia is in general large, as it includes both steppe and taiga species. The largest rodent of Mordovia is *C. fiber*, which is widely distributed in the region’s water bodies (Andreychev 2017). The rodent and insectivore fauna of Mordovia is larger than in adjacent regions. However, the fauna lacks some species that exist in adjacent regions: Ulyanovsk region - *Allocricetulus eversmanni* Brandt, 1859 (Red Book 2008); Penza region - *Spermophilus major* Pallas, 1778, *Sicista strandi* (Formozov, 1931) (Il’in et al. 2006); Nizhny Novgorod region – *Rattus rattus* (Linnaeus, 1758), *Tamias sibiricus* (Laxmann, 1769), *Pteromys volans* (Linnaeus, 1758), *Myodes rufocanus* (Sundevall, 1846) (Krivonogov et al. 2008); Chuvash region – *Tamias sibiricus* (Laxmann, 1769), *Pteromys volans* (Linnaeus, 1758) (Red Book 2010); and Ryazan region - *Rattus rattus* (Linnaeus, 1758), *Pteromys volans* (Linnaeus, 1758) (Red Book 2011). Thus, seven species of rodents and insectivores present in adjacent regions are absent from the fauna of Mordovia.

## Taxonomic coverage

The dataset contains more than 24,000 registrations of rodent and insectivore species from the districts of the Republic of Mordovia, including Saransk (Table 1, Appendix 1).

**Table 1.** Registration points of rodents and insectivores collected in the Mordovia.

Species no.	Species	Points no. (from Appendix 1)
1	*Erinaceus roumanicus*	17, 25, 31, 32, 46, 63, 75, 99, 102
2	*Erinaceus europaeus*	34, 103
3	*Talpa europaea*	1, 8, 11, 17, 24, 33, 36, 40, 44, 53, 56, 63, 64, 67, 73, 76, 81, 85, 97, 98, 102, 103, 105
4	*Desmana moschata*	68, 73, 76, 81, 95, 97, 101
5	*Sorex araneus*	6, 8, 11, 22, 24, 28, 33, 35, 40, 45, 51, 53, 56, 63, 64, 67, 73, 77, 83, 89, 98, 102, 103
6	*Sorex caecutiens*	37, 71, 67, 76, 93, 97, 102, 103
7	*Sorex minutissimus*	76
8	*Sorex isodon*	14, 37, 52, 59, 62, 66, 74, 94, 96, 102, 103
9	*Sorex minutus*	10, 11, 22, 24, 26, 33, 35, 40, 45, 51, 53, 56, 58, 63, 65, 67, 73, 77, 82, 91, 98, 102, 103
10	*Neomys fodiens*	12, 17, 33, 40, 47, 49, 53, 55, 63, 69, 73, 76, 80, 84, 86, 97, 101, 102, 103, 105
11	*Neomys anomalus*	76
12	*Crocidura suaveolens*	20, 36, 76, 102
13	*Sciurus vulgaris*	17, 31, 34, 38, 44, 56, 63, 67, 73, 76, 88, 97, 100, 102
14	*Spermophilus suslicus*	7, 31, 23, 33
15	*Marmota bobak*	1, 21, 31, 32, 39, 62
16	*Castor fiber*	1, 8, 11, 17, 24, 33, 36, 40, 44, 53, 56, 63, 64, 67, 73, 76, 81, 85, 97, 98, 102, 103, 105
17	*Dryomys nitedula*	36, 76, 102, 103
18	*Eliomys quercinus*	17, 76
19	*Glis glis*	4, 38, 42, 76, 102
20	*Muscardinus avellanarius*	17, 56, 102, 103
21	*Sicista betulina*	76, 100, 102, 103
22	*Allactaga major*	31, 33, 69, 85
23	*Spalax microphthalmus*	11, 19
24	*Myodes glareolus*	1, 8, 11, 17, 24, 33, 36, 40, 44, 53, 56, 63, 64, 67, 73, 76, 81, 85, 97, 98, 102, 103, 105
25	*Myodes rutilus*	76
26	*Arvicola amphibius*	1, 8, 11, 17, 24, 33, 36, 40, 44, 53, 56, 63, 64, 67, 73, 76, 81, 85, 97, 98, 102, 103, 105
27	*Microtus oeconomus*	24, 61, 70, 76, 92, 102, 103
28	*Cricetulus migratorius*	76
29	*Cricetus cricetus*	3, 5, 9, 16, 18, 24, 33, 37, 50, 62, 98, 104
30	*Ondatra zibethicus*	1, 8, 11, 17, 24, 33, 36, 40, 44, 53, 56, 63, 64, 67, 73, 76, 81, 85, 97, 98, 102, 103, 105
31	*Lagurus lagurus*	31, 76
32	*Microtus arvalis* s.l.	1, 8, 11, 17, 24, 33, 36, 40, 44, 53, 56, 63, 64, 67, 73, 76, 81, 85, 97, 98, 102, 103, 105
33	*Microtus agrestis*	72, 76, 87, 102, 103
34	*Microtus subterraneus*	102
35	*Apodemus agrarius*	10, 11, 22, 24, 27, 33, 35, 40, 45, 51, 53, 56, 58, 63, 65, 67, 73, 77, 82, 91, 98, 102, 103
36	*Apodemus uralensis*	1, 8, 11, 17, 24, 33, 36, 40, 44, 53, 56, 63, 64, 67, 73, 76, 81, 85, 97, 98, 102, 103, 105
37	*Apodemus flavicollis*	2, 29, 30, 33, 41, 54, 56, 57, 60, 63, 73, 78, 79, 90, 97, 103
38	*Micromys minutus*	13, 17, 31, 43, 48, 71, 76, 102
39	*Mus musculus*	10, 11, 22, 24, 27, 33, 35, 40, 45, 51, 53, 56, 58, 63, 65, 67, 73, 77, 82, 91, 98, 102, 103
40	*Rattus norvegicus*	1, 8, 11, 17, 24, 33, 36, 40, 44, 53, 56, 63, 64, 67, 73, 76, 81, 85, 97, 98, 102, 103, 105

## Taxonomic ranks

**Kingdom**: Animalia

**Phylum**: Chordata

**Class**: Mammalia

**Order**: Eulipotyphla, Rodentia

**Family**: Erinaceidae, Talpidae, Soricidae, Sciuridae, Castoridae, Gliridae, Sminthidae, Allactagidae, Spalacidae, Cricetidae, Muridae

**Genus**: *Talpa*, *Desmana*, *Sorex*, *Neomys*, *Crocidura*, *Sciurus*, *Spermophilus*, *Marmota*, *Castor*, *Dryomys*, *Eliomys*, *Glis*, *Muscardinus*, *Sicista*, *Allactaga*, *Spalax*, *Myodes*, *Arvicola*, *Microtus*, *Cricetulus*, *Cricetus*, *Ondatra*, *Lagurus*, *Microtus*, *Apodemus*, *Micromys*, *Mus*, *Rattus*

**Species**: *Erinaceus roumanicus*, *Erinaceus europaeus*, *Talpa europaea*, *Desmana moschata*, *Sorex araneus*, *Sorex caecutiens*, *Sorex minutissimus*, *Sorex isodon*, *Sorex minutus*, *Neomys fodiens*, *Neomys anomalus*, *Crocidura suaveolens*, *Sciurus vulgaris*, *Spermophilus suslicus*, *Marmota bobak*, *Castor fiber*, *Dryomys nitedula*, *Eliomys quercinus*, *Glis glis*, *Muscardinus avellanarius*, *Sicista betulina*, *Allactaga major*, *Spalax microphthalmus*, *Myodes glareolus*, *Myodes rutilus*, *Arvicola amphibius*, *Microtus oeconomus*, *Cricetulus migratorius*, *Cricetus cricetus*, *Ondatra zibethicus*, *Lagurus lagurus*, *Microtus arvalis*, *Microtus rossiaemeridionalis*, *Microtus agrestis*, *Microtus subterraneus*, *Apodemus agrarius*, *Apodemus uralensis*, *Apodemus flavicollis*, *Micromys minutus*, *Mus musculus*, *Rattus norvegicus*

## Spatial coverage

The dataset covers the entire Republic of Mordovia within 53°38'N to 55°11'N and 42°11'E to 46°45'E.

## Temporal coverage

The data were collected from 2006 to 2020.

## Method

Most of the dataset was obtained in the Republic of Mordovia during expedition research and at the biological station. The work is based on the materials of our surveys of rodents and insectivorous mammals conducted in the Republic of Mordovia, using trap lines and pitfall arrays using traditional methods. Small rodents were captured using small spring snap-traps (120 × 55 mm) left over night in lines of from 50 to 100 traps with a distance of 5 m between them and baited with bread and sunflower oil. We also used live traps baited with salami and apple to catch dormice. Voucher specimens are stored in the personal collection A. Andreychev, Saransk (teriomordovia@bk.ru). Data on *Erinaceus roumanicus*, *Erinaceus europaeus*, *Talpa europaea*, *Desmana moschata*, *Sciurus vulgaris*, *Spermophilus suslicus*, *Marmota bobak*, *Castor fiber*, *Allactaga major*, *Spalax microphthalmus*, *Cricetus cricetus*, and *Ondatra zibethicus* were obtained via direct observations, recording and/or detection of the traces of their activities (tracks, burrows, etc.). Latin names of species are given according to the classical nomenclature (Wilson and Reeder 2005).

## Appendix 1

**Table:** Points of the Republic of Mordovia in which mammals are recorded.

Points no.	District	Location	Geographic coordinates
1	Lyambirskiy	Atemar	54.0980°N, 45.2138°E
2	Kochkurovskiy	Vorob’evka	54.0251°N, 45.1754°E
3		Vnukovka	54.0118°N, 45.1609°E
4		Novotyaglovka	53.5506°N, 45.1778°E
5	Ichalkovskiy	Lobaski	54.3662°N, 45.1007°E
6	Lyambirskiy	Cheremishevo	54.1579°N, 45.0604°E
7	Romodanovskiy	Malaya Chfarovka	54.2435°N, 45.1750°E
8		Romodanovo	54.2494°N, 45.2043°E
9		Kavtorovka	54.2187°N, 45.1491°E
10		Kurilovo	54.2854°N, 45.2805°E
11	Ruzaevskiy	Levzhenskij	54.0618°N, 45.0517°E
12		Popovka	54.0690°N, 45.0310°E
13		Klyucharevo	54.0846°N, 45.0121°E
14		Ruzaevka	54.0246°N, 44.5609°E
15		Bogolyubovka	54.0596°N, 44.5238°E
16		Tatarskij Shebdas	53.5851°N, 44.5444°E
17	Saransk	Saransk	54.1328°N, 45.1102°E
18		Zykovo	54.0495°N, 45.0589°E
19		Dobrovol’nyj	54.0807°N, 45.0506°E
20		Pushkino	54.0815°N, 45.1151°E
21		Makarovka	54.1037°N, 45.1717°E
22		Kulikovka	54.0689°N, 45.1217°E
23	Ardatovskiy	Zharenki	54.4420°N, 46.1421°E
24	Atyashevskiy	Tarasovo	54.3578°N, 46.1334°E
25	Chamzinskiy	Rep’evka	54.2468°N, 45.4177°E
26		Gorbunovka	54.3030°N, 45.5188°E
27		Azar’evka	54.3367°N, 45.3987°E
28		Komsomol’skij	54.2672°N, 45.5183°E
29		Lyulya	54.2755°N, 45.5589°E
30	Ichalkovskiy	Vechkusy	54.4196°N, 45.3641°E
31	Bol’shebereznikovskiy	Simkino	54.1527°N, 46.1168°E
32	Dubenskiy	Engalychevo	54.1846°N, 46.2649°E
33		Nikolaevka	54.2105°N, 46.3065°E
34		Purkaevo	54.2316°N, 46.3625°E
35	Kochkurovskiy	Novaya Pyrma	54.0017°N, 45.2922°E
36	Chamzinskiy	Chamzinka	54.2318°N, 45.4739°E
37		Medaevo	54.2593°N, 46.0009°E
38		Pyangelej	54.1778°N, 45.4172°E
39		Picheury	54.1847°N, 45.4883°E
40	Insarskiy	Luhmenskij Majdan	53.4460°N, 44.1146°E
41		Potulovka	53.4215°N, 44.2515°E
42	Kadoshkinskiy	Adashevo	53.5712°N, 44.1884°E
43	Kovylkinskiy	Mordovskoe Kolomasovo	53.5837°N, 44.0684°E
44	Atyur’evskiy	Shustruj	54.1441°N, 43.2251°E
45		Kurtashki	54.1916°N, 43.2639°E
46	Zubovo-Polyanskiy	Achadovo	53.5385°N, 43.0009°E
47	Kovylkinskiy	Gumny	54.0067°N, 43.4337°E
48	Krasnoslobodskiy	Zheltonogovo	54.2875°N, 43.4456°E
49		Slobodskie Dubrovki	54.2483°N, 43.3468°E
50	Temnikovskiy	Bulaevo	54.3331°N, 43.3354°E
51	Torbeevskiy	Salazgor’	54.0718°N, 43.0735°E
52		Drakino	54.0269°N, 43.1445°E
53	Kadoshkinskiy	Pushkino	54.0528°N, 44.2329°E
54	Ruzaevskiy	Boldovo	53.5981°N, 44.3908°E
55	Staroshajgovskiy	Mal’keevka	54.2428°N, 44.4723°E
56		Staraya Terizmorga	54.1569°N, 44.3043°E
57	Lyambirskiy	Dal’nij	54.2883°N, 44.5892°E
58		Yazykovo	54.2680°N, 44.5864°E
59		Lopatino	54.1738°N, 45.0136°E
60	Ardatovskiy	Kurakino	54.5645°N, 46.0831°E
61		Staroe Ardatovo	54.5843°N, 46.1336°E
62	Bol’sheignatovskiy	Petrovka	54.5854°N, 45.2930°E
63		Kirzhemany	54.5850°N, 45.4432°E
64	Kovylkinskiy	Rybkino	54.1531°N, 43.4799°E
65		Kovylyaj	54.1016°N, 43.5071°E
66	Krasnoslobodskiy	Krasnaya Podgora	54.2887°N, 43.4870°E
67		Lesnoj	54.2613°N, 43.5214°E
68		Zarechnoe	54.2469°N, 43.5124°E
69		Staraya Ryabka	54.2050°N, 43.5046°E
70		Samozlejka	54.1829°N, 43.4931°E
71	El’nikovskiy	Churino	54.3995°N, 43.4471°E
72		Russkie Poshaty	54.4078°N, 43.4436°E
73		Starye Pichingushi	54.3391°N, 43.5029°E
74	Temnikovskiy	Staryj Gorod	54.4175°N, 43.0609°E
75		Temnikov	54.3748°N, 43.1139°E
76		MGZ im. P.G. Smidovicha	54.4286°N, 43.1423°E
77		Kozlovka	54.3816°N, 43.2114°E
78		Sosnovka	54.4281°N, 43.1683°E
79	Ten’gushevskiy	Standrovo	54.3953°N, 42.3943°E
80		Shelubej	54.4074°N, 42.4291°E
81		Telimerki	54.4364°N, 42.4580°E
82		Feklisov	54.4289°N, 42.5073°E
83		Vedenyapino	54.4416°N, 42.5857°E
84	Atyur’evskiy	Arga	54.2034°N, 43.0912°E
85	Zubovo-Polyanskiy	Podlyasovo	54.1941°N, 42.4184°E
86		Svezhen’kaya	54.0035°N, 42.2674°E
87		Izvest’	53.5609°N, 42.2746°E
88		Vysha	53.5033°N, 42.2320°E
89		Gorodishche	53.4553°N, 42.2921°E
90		Zhukovka	53.5293°N, 42.4358°E
91		Shiringushi	53.5123°N, 42.4571°E
92		Ozernyj	54.2510°N, 42.4141°E
93		Lesnoj	54.2774°N, 42.4323°E
94		Romanovka	54.1481°N, 42.4416°E
95	Zubovo-Polyanskiy	Leplej	54.1883°N, 42.4890°E
96	Ten’gushevskiy	Yuzga	54.3277°N, 42.5919°E
97	Torbeevskiy	Vindrej	54.1554°N, 42.5523°E
98	Ardatovskiy	Turgenevo	54.5150°N, 46.1648°E
99		Redkodub’e	54.4743°N, 46.0990°E
100		Lun’ga	54.4864°N, 45.5652°E
101		Lun’ginskij Majdan	54.5046°N, 45.4821°E
102	Ichalkovskiy	Smol’nyj	54.4592°N, 45.3690°E
103	Bol’shebereznikovskiy	Biologicheskaya stanciya MGU	54.1014°N, 46.0988°E
104		Special’nyj	54.0262°N, 45.5324°E
105	Kochkurovskiy	Mordovskoe Davydovo	53.5856°N, 45.4535°E
